# The expression status and clinical value of TIM-3 in intrahepatic cholangiocarcinoma

**DOI:** 10.1186/s12885-026-16292-9

**Published:** 2026-06-05

**Authors:** Ziwen Yin, Jiexu Li, Le Liu, Lin Zhang, Wei Zhang, Bingqi Ma

**Affiliations:** 1 Department of Hepatopancreatobiliary Surgery, Affiliated Hospital of Shandong Second Medical University, Weifang, China; 2 Department of Hepatobiliary Surgery, Tianjin Medical University Cancer Institute and Hospital, National Clinical Research Center for Cancer, Key Laboratory of Cancer Prevention and Therapy, Tianjin’s Clinical Research Center for Cancer, Tianjin, China

**Keywords:** T cell immunoglobulin and mucin-domain containing protein 3 (TIM-3), Intrahepatic cholangiocarcinoma (ICC), Prognosis, Immune checkpionts, Tumor-infiltrating lymphocytes (TILs), Immunology

## Abstract

**Background:**

T cell immunoglobulin and mucin-domain containing protein 3 (TIM-3) is an immune checkpoint that plays a crucial role in immune exhaustion. High expression of TIM-3 has been implicated in exerting immunosuppression across a variety of malignant tumors. Elucidating the expression profile of TIM-3 and its prognostic impact may hold great significance for the therapeutic management of intrahepatic cholangiocarcinoma (ICC).

**Methods:**

We analyzed 117 ICC patients using immunohistochemical staining for TIM-3 and other immune checkpoints to examined their expression and immune cell infiltration in ICC tissue samples. Furthermore, the correlation of checkpoint expression with clinical characteristics and prognosis were analyzed.

**Results:**

High expression of TIM-3 in tumor cells was observed in 61 patients (52.1%) and was significantly correlated with poorer tumor differentiation (*P* = 0.019). Survival analysis showed that high TIM-3 expression in tumor cells was a prognostic factor for disease-free survival (DFS) and overall survival (OS) in univariate analysis, and an independent risk predictor for DFS in multivariate analysis (*P* = 0.048, HR = 1.589, 95%CI = 1.004–2.515). Furthermore, TIM-3 expression in ICC tissues was significantly correlated with CD4⁺ and CD8⁺ tumor-infiltrating lymphocytes (both *P* < 0.005).

**Conclusion:**

Patients with high TIM-3 expression in tumor cells had shorter DFS and OS, which could serve as a valuable biomarker for predicting the inflammatory status and prognosis of ICC. Targeting TIM-3 may represent a promising therapeutic strategy for ICC.

## Introduction

Intrahepatic cholangiocarcinoma (ICC) is a highly lethal, aggressive, and heterogeneous malignancy of the biliary ducts [1]. The incidence of ICC has been on the rise globally in recent years [2]. However, due to its nonspecific symptoms, approximately 60–70% of cases are at an unresectable advanced stage when they are diagnosed [3]. Since the ABC-02 trial, gemcitabine plus cisplatin (GC) has become the standard first-line regimen with the median overall survival (mOS) of 11.7 months, which remains unsatisfactory for patients with advanced ICC [4, 5]. Immune checkpoint inhibitors (ICIs) have achieved remarkable efficacy in the treatment of various malignancies and have been incorporated into standard therapeutic strategies [6–8]. The TOPAZ-1 and KEYNOTE-966 trials further established ICIs combined chemotherapy as a novel first-line treatment for ICC, but only improved mOS by 1.5 months and 1.8 months, respectively [9, 10]. These findings confirm the clinical value of immunotherapy, while also indicating that current therapeutic strategies are still far from meeting clinical needs.

Immune evasion is a hallmark feature of cancer progression. ICC is characterized by a prominent desmoplastic stroma, which is rich in immune cells such as cancer-associated fibroblasts (CAFs) and tumor-associated macrophages (TAMs) [11]. These cells collectively contribute to the formation of an immunosuppressive tumor microenvironment (TME) by promoting immune tolerance, tumor proliferation, and metastasis. Immune checkpoints expressed on both tumor cells and infiltrating immune cells also play critical roles in this progression. T cell immunoglobulin and mucin-domain containing protein 3 (TIM-3) is one of transmembrane immune checkpoint proteins. Previous Studies have revealed that TIM-3 is also expressed on tumor cells in multiple malignancies including advanced non-small cell lung cancer (NSCLC), gastric cancer (GC), and hepatocellular carcinoma (HCC), and its differential expression is closely associated with patient prognosis across different cancers [12–14]. However, the cell-specific prognostic value of TIM-3 in ICC, as well as its distinct interaction with the TME, remains unclear. Accordingly, this study aimed to analyze the correlation between TIM-3 expression and clinicopathological characteristics, inflammatory status, and its prognostic value in the prognosis of ICC.

## Materials and methods

### Patients and tissue samples

A total of 127 ICC patients who underwent surgical resection at Tianjin Medical University Cancer Institute and Hospital, along with 32 patients from the Affiliated Hospital of Shandong Second Medical University, were retrospectively enrolled in this study from 2018 to 2024. Patient recruitment, inclusion/exclusion criteria, and follow-up protocols were consistent with our previous study with minor adjustments [15]. To ensure the rigor of the study, patients who died within a short period after surgery (≤ 30 days) due to surgical complications or other causes unrelated to the tumor, as well as patients for whom complete prognostic information could not be obtained, were excluded.

After preliminary screening, a total of 42 patients were excluded (including 13 cases with atypical ICC diagnosis, 16 cases with non-R0 resection, 10 cases lost to follow-up within a short period, 1 case died of postoperative complications, and 2 cases died of non-tumor-related causes). Ultimately, 117 patients were included for statistical analysis. All patients were followed up every 3 months to monitor tumor recurrence. DFS was defined as the interval from surgery to first recurrence or last follow-up, whereas OS was defined as the interval from diagnosis to death or last follow-up.

### Ethics approval and consent to participate

The Ethics Committee of Tianjin Medical University Cancer Institute and Hospital (bc2019026) and the Medical Ethics Committee of the Affiliated Hospital of Shandong Second Medical University approved this study (wyfy-2022-ky-196). Written informed consent was obtained from all patients prior to their participation in the study. This study was conducted in accordance with the Declaration of Helsinki and Good Clinical Practice (GCP) guidelines.

### Public dataset exploration

Expression of HAVCR2 (also known as TIM-3) and corresponding clinical information of ICC patients and normal control individuals were downloaded from The Cancer Genome Atlas (TCGA) and Gene Expression Omnibus (GEO) databases. The expression pattern of TIM-3 in ICC was analyzed based on the TCGA-CHOL cohort, GSE89749 and GSE107943 dataset.

### Immunohistochemistry (IHC)

Tissue microarrays (TMAs, 4 μm thick, 2 mm diameter) were constructed using representative tumor and peritumoral regions, as previously described [15]. IHC staining was performed using antibodies against TIM-3 (ab241332, 1:200, Abcam), LAG-3 (ab209236, 1:500, Abcam), PD-1 (ab137132, 1:500, Abcam), PD-L1 (ab228415, 1:500, Abcam).

**Fig. 1:**
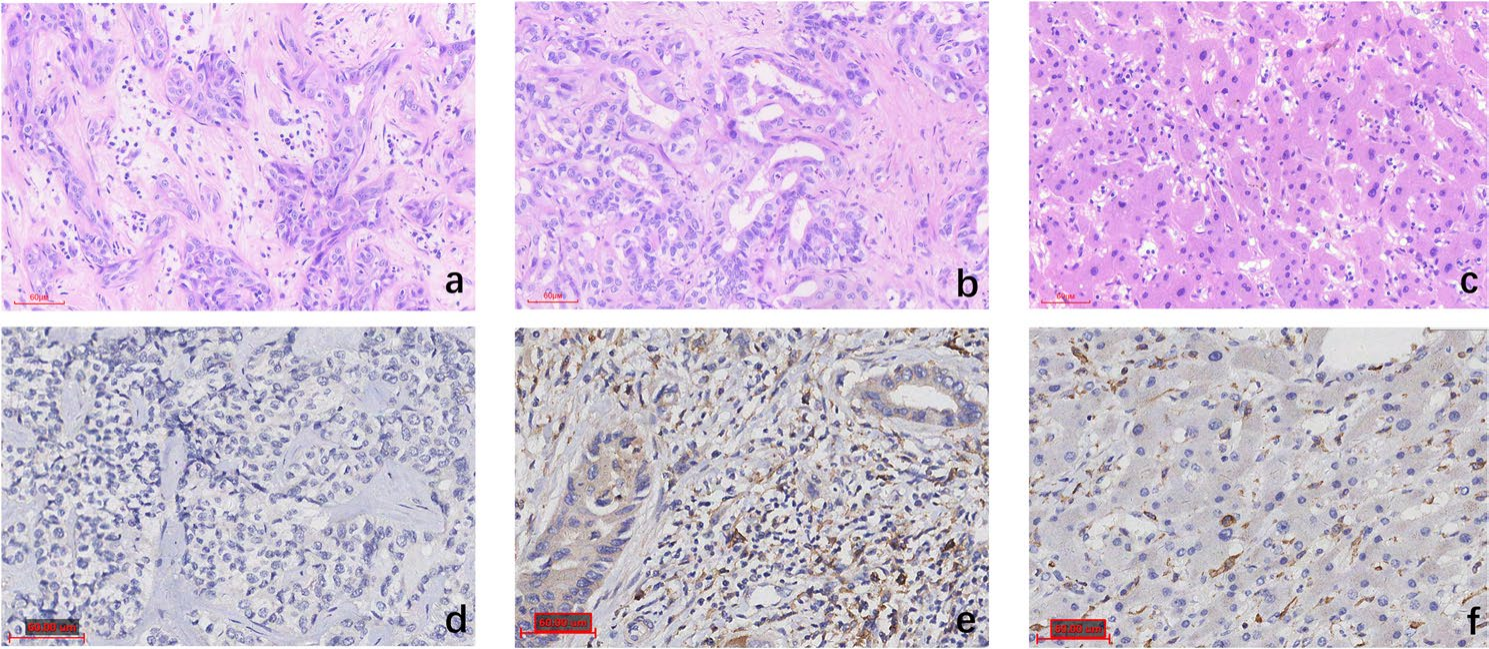
TIM-3 expression on tumor tissue and adjacent normal specimen evaluated by IHC and HE staining. **a** and **d** Low TIM-3 expression in ICC. **b** and **e** High TIM-3 expression in ICC. **c** and **f** Negative TIM-3 expression in normal liver cells (e.g., biliary epithelial cells). Abbreviations: TIM-3, T cell immunoglobulin and mucin-domain containing protein 3; IHC, Immunohistochemistry; HE, hematoxylin-eosin

TIM-3 was observed in the cytoplasm and cell membrane and was evaluated both in tumor cells and tumor-infiltrating lymphocytes (TILs). In tumor cells, the assessment of TIM-3 expression using semi-quantitative assessment was performed in accordance with methods described in previous studies [15]. In TILs, the TIM-3 expression was defined as the percentage of immune cells with a positive punctate, membrane, and/or cytoplasmic staining relative to all immune cells in the tumor region containing at least 100 viable tumor cells. Immune cells with TIM-3 ≥ 1% were considered TIM-3-positive. The programmed death-ligand 1 (PD-L1) combined positive score (CPS) was defined as the percentage of PD-L1-stained cells, including tumor cells, lymphocytes, and macrophages, divided by the total number of viable tumor cells and multiplied by 100. PD-L1 positivity was defined as PD-L1 CPS ≥ 1% (Fig. 1). Other molecular detection methods were performed according to previous studies [15, 16]. All scoring was independently performed by two investigators blinded to clinical data.

### Statistical and data analysis

Chi-square test or Fisher’s exact test was used for categorical variables, and the t-test or Mann-Whitney U test for continuous variables. Survival analysis was performed using the Kaplan-Meier method, and the Log-rank test was used to compare differences in DFS and OS between subgroups. The prognostic value of clinical factors was analyzed using the Cox proportional hazards model, adjusting for bias caused by tumor characteristics (e.g., TNM staging). Variables with P value < 0.1 in univariate analysis and established prognostic factors were included in multivariate Cox model. Spearman’s correlation analysis and Fisher’s exact test were used to assess the association between TIM-3 and other immune checkpoints, while Cohen’s kappa test was applied to evaluate the concordance between TIM-3 and PD-L1 expression. All analyses were performed using SPSS software version 27 (IBM Corp., Armonk, NY, USA). A two-tailed P value < 0.05 was considered statistically significant.

## Results

### Patient characteristics

The study cohort included 53 females (45.3%) and 64 males (54.7%), with a median age of 59 years (range 28–77 years). Baseline demographics are summarized in Table 1. The mOS and mDFS were 39.2 months (95% confidence interval [95% CI]: 33.0-45.4 months) and 15.2 months (95% CI: 10.9–19.4 months), respectively.

**Table 1:** Baseline clinicopathological characteristics of enrolled ICC patients

Variables	Category	Numbers(*n* = 117)	Percentage (%)
Sex	Male	64	54.7
	Female	53	45.3
Age(years)	Median (range)	59 (28–77)	--
	< 60	63	53.8
	≥ 60	54	46.2
T category	T1-T2	100	85.5
	T3-T4	17	14.5
N category	N0	87	74.4
	N1	30	25.6
M category	M0	102	87.2
	M1	15	12.8
TNM stage	I-II	82	70.1
	III-IV	35	29.9
Postoperative recurrence	No	39	33.3
	Yes	78	66.7
Differentiation grade	Poor	54	53.8
	Well or moderate	63	46.2
Tumor diameter (cm)	< 5	66	56.4
	≥ 5	51	43.6
History of hepatitis B	No	40	34.2
	Yes	77	65.8
Cirrhosis	No	78	66.7
	Yes	39	33.3
Neu (x10^9^/l)	< 5.0	93	79.5
	≥ 5.0	24	20.5
Lym (x10^9^/l)	< 2.0	77	65.8
	≥ 2.0	40	34.2
NLR	< 3.0	94	80.3
	≥ 3.0	23	19.7
CEA (µg/l)	< 5	91	77.8
	≥ 5	26	22.2
CA19-9 (U/ml)	< 39	60	51.3
	≥ 39	57	48.7
TIM-3 expression (tumor cells)	Low	56	47.9
	High	61	52.1
TIM-3 expression (TILs)	Low	66	56.4
	High	51	43.6
PD-L1 expression	low	74	63.2
	high	43	36.8

### Prognostic significance of TIM-3 in patients with ICC

Database analysis revealed that the expression of HAVCR2 was significantly higher in ICC tissues than in adjacent normal liver tissues (all *P* < 0.01 and log_2_FC > 1), with consistent trends observed across the three databases. We further confirmed the expression profile of TIM-3 and its impact on postoperative clinical prognosis in 117 ICC patients.

**Fig. 2:**
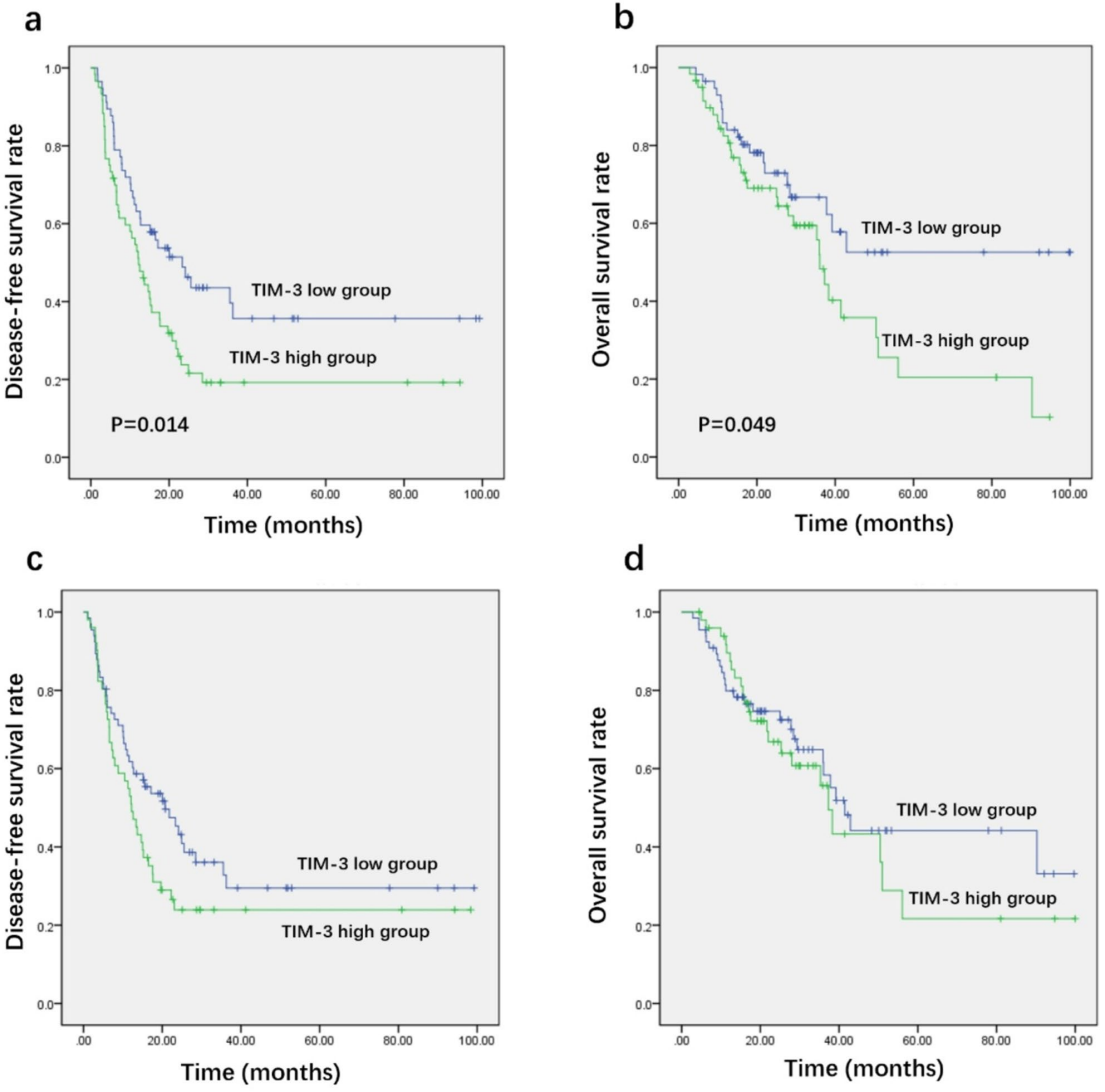
Kaplan‑Meier curves for patients with ICC. Patients with high expression of TIM-3 in tumor cells had shorter (**a**) disease‑free survival and (**b**) overall survival. No significant differences were observed in DFS (**c**) or OS (**d**) between patients with high and low TIM‑3 expression in TILs

During the follow-up period, a total of 78 patients experienced tumor recurrence, and 50 patients died or were lost to follow-up. Survival analysis revealed 3-year DFS rate and 3-year OS rate of patients in this cohort were 25.6% and 48.7%, respectively. Univariate analysis indicated that patients with high TIM-3 expression in tumor cells had significantly shorter DFS (*P* = 0.014) and OS (*P* = 0.049) compared with those with low TIM-3 expression in tumor cells. However, no statistically significant differences in DFS (*P* = 0.097) and OS (*P* = 0.507) were observed between patients with high and low TIM-3 expression in TILs (Fig. 2). In addition, neutrophil-to-lymphocyte ratio (NLR), CEA, CA19-9 levels, T stage, and M stage were identified as prognostic factors for DFS in ICC patients. Multivariate analysis included these five factors that high TIM-3 expression in tumor cells was a favorable independent predictor of DFS in ICC (*P* = 0.048, HR = 1.589, 95% CI: 1.004–2.515). However, TIM-3 expression only showed a trend toward being an independent prognostic factor for OS, as the correlation with OS did not reach statistical significance (Table 2; Fig. 2).

**Table 2:** Univariate and multivariate analysis for prognostic factors in ICC

Variables	DFS			
	Univariate	Multivariate		
	*P*-value	HR	95%CI	*P*-value
TIM-3 expression in tumor cells, low vs. high	0.014	1.589	1.004–2.515	0.048
T category, T1-T2 vs. T3-T4	0.005	1.284	0.666–2.474	0.455
M category, M0 vs. M1	0.000	2.364	1.183–4.723	0.015
CEA, < 5 vs. ≥5	0.002	2.026	1.192–3.442	0.009
CA199, < 39 vs. ≥39	0.002	1.252	0.753–2.080	0.386
NLR, < 3 vs. ≥3	0.000	2.300	1.279–4.138	0.005

*DFS*  disease-free survival, *NLR*  neutrophil-lymphocyte ratio, *TIM-3*  T-cell immunoglobulin and mucin domain-containing protein 3

### Association between TIM-3 expression and clinical characteristics in patients with ICC

Patients with high TIM-3 expression in tumor cells had poorer tumor differentiation (*P* = 0.019) and higher postoperative recurrence rate (*P* = 0.019). Similarly, patients with high TIM-3 expression in lymphocytes had smaller tumor diameters (*P* = 0.007) and poorer tumor differentiation (*P* = 0.016). High TIM-3 expression in both cell populations was significantly associated with high PD-L1 expression (*P* = 0.022 in tumor cells and *P* < 0.001 in TILs, Table 3).

**Table 3:** Association between TIM-3 expression and clinicopathological characteristics in patients with ICC

		TIM-3 expression in tumor cells			TIM-3 expression in TILs		
Variables	numbers	low	high	*P*-value	low	high	*P*-value
Sex				0.947			0.431
Male	64	31	33		34	30	
Female	53	26	27		32	21	
Age(years)				0.110			0.195
< 60	63	35	28		39	24	
≥ 60	54	22	32		27	27	
T category				0.367			0.456
T1-T2	100	47	53		55	45	
T3-T4	17	10	7		11	6	
N category				0.871			0.412
N0	87	42	45		51	36	
N1	30	15	15		15	15	
M category				0.702			0.170
M0	102	49	53		60	42	
M1	15	8	7		6	9	
TNM stage				0.431			0.478
I-II	82	38	44		48	34	
III-IV	35	19	16		18	17	
Postoperative recurrence				0.019			0.114
No	39	25	14		26	13	
Yes	78	32	46		40	38	
Differentiation grade				0.019			0.016
Poor	54	20	34		24	30	
Well or moderate	63	37	26		42	21	
Tumor diameter (cm)				0.954			0.007
< 5	66	32	34		30	36	
≥ 5	51	25	26		36	15	
History of hepatitis B				0.078			0.177
No	40	24	16		26	14	
Yes	77	33	44		40	37	
Cirrhosis				0.050			0.114
No	78	43	35		48	30	
Yes	39	14	25		18	21	
Neu (x10^9^/l)				0.888			0.110
< 5.0	93	45	48		49	44	
≥ 5.0	24	12	12		17	7	
Lym (x10^9^/l)				0.849			0.572
< 2.0	77	38	39		42	35	
≥ 2.0	40	19	21		24	16	
NLR				0.575			0.990
< 3.0	94	47	47		53	41	
≥ 3.0	23	10	13		13	10	
CEA (µg/l)				0.458			0.100
< 5	91	46	45		55	36	
≥ 5	26	11	15		11	15	
CA19-9 (U/ml)				0.113			0.180
< 39	60	34	27		38	23	
≥ 39	56	23	33		28	28	
PD-L1 expression				0.022			0.000
low	74	42	32		53	21	
high	43	15	28		13	30	

*NLR* neutrophil-lymphocyte ratio, *TIM-3* T-cell immunoglobulin and mucin domain-containing protein 3, *PD-L1* programmed cell death ligand 1

**Fig. 3:**
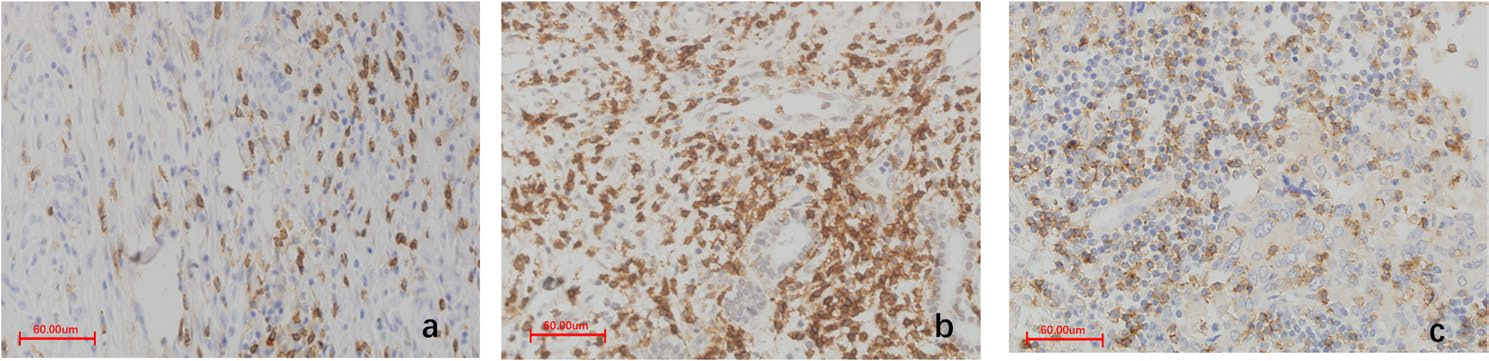
Increased tumor-infiltrating T lymphocytes in iCC patients with high TIM-3 expression. **a** Representative staining of CD4⁺ TILs. **b** Representative staining of CD8⁺ TILs. **c** Representative staining of CD45RO⁺ TILs

**Fig. 4:**
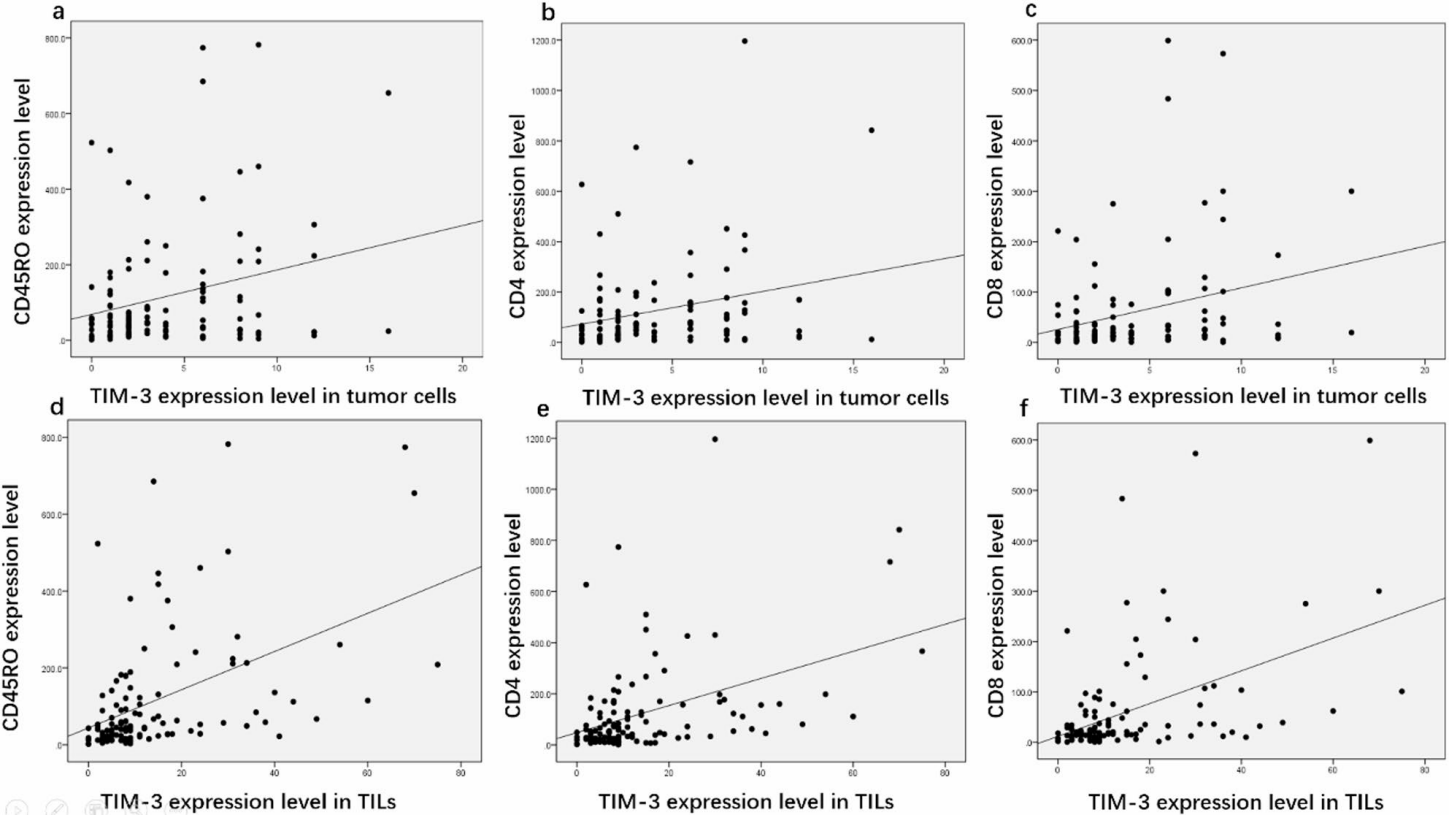
Correlation between TIM-3 Expression and TILs Infiltration in ICC. Correlations of high TIM‑3 expression in tumor cells with CD45RO⁺ (**a**), CD4⁺ (**b**), and CD8⁺ (**c**), and of high TIM‑3 expression in TILs with CD45RO⁺ (**d**), CD4⁺ (**e**), and CD8⁺ (**f**)

To explore the potential mechanism underlying the prognostic value of TIM-3 in ICC, we further investigated the subtypes of TILs in ICC tumor tissues by IHC (Table 4; Figs. 3 and 4). The results showed that patients with high TIM-3 expression in tumor cells had significantly increased infiltration levels of CD4⁺ (*P* = 0.007) and CD8⁺ (*P* = 0.021) TILs. In contrast, patients with high TIM-3 expression in TILs exhibited significantly elevated infiltration levels of CD4⁺, CD8⁺, and CD45RO⁺ TILs (all *P* < 0.001). Spearman correlation analysis revealed that high TIM-3 expression in tumor cells was positively correlated with the infiltration proportions of CD4⁺ (Spearman rₛ=0.276, *P* = 0.003) and CD8⁺ (Spearman rₛ=0.291, *P* = 0.001) TILs. High TIM-3 expression in TILs was moderately positively correlated with the infiltration levels of CD45RO⁺ (Spearman rₛ=0.488, *P* < 0.001), CD4⁺ (Spearman rₛ=0.416, *P* < 0.001), and CD8⁺ (Spearman rₛ=0.421, *P* < 0.001) TILs.

**Table 4:** Correlation between TIM-3 Expression and TILs Infiltration in Patients with ICC

		TIM-3 expression in tumor cells			TIM-3 expression in TILs		
Variables	numbers	low	high	*P* value	low	high	*P* value
CD45RO				0.305			0.000
low	60	32	28		47	13	
high	57	25	32		19	38	
CD4				0.007			0.000
low	59	36	23		44	15	
high	58	21	37		22	36	
CD8				0.021			0.000
low	57	34	23		43	14	
high	60	23	37		23	37	

### Correlation between TIM-3 and other immune checkpoint molecules

**Fig. 5:**
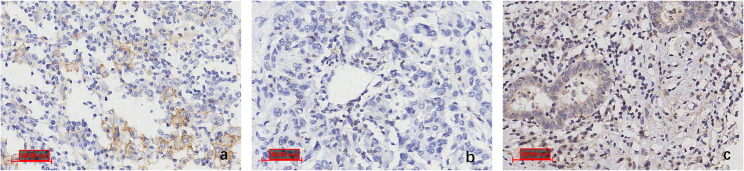
Representative IHC staining of other checkpoint expressions in ICC. **a** High PD-1 expression in ICC. **b** High PD-L1 expression in ICC. **c** High LAG-3 expression in ICC

We further examined the expression of other immune checkpoints in tumor tissues from 117 ICC patients (Fig. 5). High TIM-3 expression in tumor cells was positively correlated with high expressions of PD-1(Spearman rₛ=0.275, *P* = 0.003), PD-L1 (Spearman rₛ=0.317, *P* = 0.001) and LAG-3 (Spearman rₛ=0.242, *P* = 0.009), suggesting a synergistic expression relationship among these immune checkpoints. In the entire study cohort, 36.8% of specimens had a PD-L1 combined positive score (CPS) ≥ 1. 45.9% of LAG-3-high expression tumor cells were PD-L1 CPS ≥ 1, whereas 58.8% of LAG-3-low expression TILs presented PD-L1 CPS ≥ 1. Although high TIM-3 expression in tumor cells was statistically correlated with high PD-L1 expression (*P* = 0.022), the consistency of their expression was low (Kappa = 0.202, 95% CI: 0.024–0.380). In addition, the co-expression of TIM-3 and PD-L1 was not significantly correlated with OS or DFS in ICC patients.

## Discussion

In the present study, we identified divergent expression levels of TIM‑3 in tumor cells and TILs. High TIM‑3 expression was observed in tumor cells in 52.1% (61/117) of patients and in TILs in 43.6% (51/117) of patients. Notably, the prognostic significance of TIM‑3 expression also differed between these two cell types. Univariate analysis showed that high TIM‑3 expression in tumor cells was associated with shorter DFS (*P* = 0.014) and OS (*P* = 0.049). Multivariate analysis further confirmed that high TIM‑3 expression in tumor cells was an independent risk factor for DFS (*P* = 0.048, HR = 1.589, 95% CI: 1.004–2.515). These findings are consistent with the negative regulatory role of TIM‑3 in T‑cell activation reported in other studies. Meta-analysis has showed that higher TIM‑3 expression was significantly associated with short OS (*P* < 0.001, HR = 1.89, 95% CI: 1.38–2.57) and more advanced tumor stage (*P* < 0.001, III/IV vs. I/II, RR = 2.02; 95% CI: 1.45–2.81) in HCC [13].

From the perspective of clinical characteristics, as a negative regulatory factor, the function of TIM-3 seems to be more effective on tumor cells. Patients with high TIM‑3 expression in tumor cells exhibited poorer tumor differentiation (*P* = 0.019), which proves our survival analysis form another side. TIM‑3 is expressed in tumor cells of various malignancies and promotes immune evasion by inhibiting CD4⁺ T cell activation to block Th1 polarization and reduce tumor cell adhesion via the IL‑6‑STAT3 pathway, or regulating epithelial‑mesenchymal transition (EMT) through downregulating E‑cadherin and upregulating N‑cadherin [17]. On the other hand, ligands of TIM-3 are widely distributed in the TME, which may mediate crosstalk between tumor cells and non-parenchymal cells, further influencing the aggressive phenotype of tumor cells [18]. However, high TIM‑3 expression in TILs showed no statistically significant association with DFS (*P* = 0.097) or OS (*P* = 0.507), suggesting limited prognostic value in ICC. TIM-3 expressed on TILs appears to exert distinct functions. TIM-3 interacts with multiple ligands, including GAL-9 [19], PtdSer [20], HMGB1 [21], and CEACAM1 [22]. Upon binding to GAL-9, TIM-3 expressed on CD4^+^ and CD8^+^ TILs induces intracellular Ca²⁺ flux, leading to Th1 cell apoptosis. Alternatively, TIM-3 can form a heterodimer with CEACAM1 to mediate T-cell exhaustion and induce immune tolerance. Furthermore, TIM-3 is also involved in the immunosuppressive function of Tregs [23]. High expression of TIM-3 in TILs was associated with tumor size and differentiation grade, indirectly indicating its immunosuppressive role in the TME.

From the perspective of the immune characteristics of ICC, Job et al. classified ICC into four immune subtypes, with 11% categorized as the immune-inflamed subtype, characterized by abundant T-cell infiltration and activated immune checkpoint pathways [24]. In such patients, TILs are enriched, and the likelihood of TIM‑3 expression is relatively higher than other subtypes. In this study, the low proportion of patients with high lymphocyte infiltration in the present study is consistent with that reported by Job et al. Moreover, high TIM-3 expression in both tumor cells and TILs was significantly associated with increased TIL infiltration. However, this observation may merely reflect concomitant immune activation within the ICC microenvironment and could be influenced by baseline immune infiltration levels across distinct ICC immune subtypes. Further functional validation is warranted to elucidate the underlying mechanisms.

Additionally, although our study identified the correlation between PD‑L1 and TIM‑3 expression, their co‑expression was not a predictive factor of DFS or OS. Crosstalk between different immune checkpoints provides a rationale for dual or combination immunotherapy [25, 26]. However, further clinical trials are warranted to explore the combination of anti‑TIM‑3 monoclonal antibodies with other ICIs. Our findings highlight the potential synergistic effect of TIM‑3 in ICC. Therefore, chemotherapy combined with anti‑TIM‑3, anti‑PD‑1, or other monoclonal antibodies may represent a promising first‑line strategy for advanced ICC in future clinical trials.

This study has several limitations. First, the small sample size from two institutions may increase the risk of false‑negative results in statistical analyses. Second, as a post hoc biomarker analysis, this study is subject to inherent biases of retrospective research. Although we confirmed the prognostic association of TIM‑3 expression, the molecular mechanism remains to be fully elucidated and requires further validation using in vitro cellular assays and in vivo animal models.

## Conclusions

In conclusion, we identified a striking difference in the prognostic value of TIM‑3 between tumor cells and TILs in ICC. High TIM‑3 expression in tumor cells, rather than in TILs, serves as an independent and effective prognostic biomarker for ICC patients and is closely associated with clinicopathological features such as differentiation grade. TIM‑3 represents a potential candidate for future immunotherapy development, but requires further preclinical and clinical validation.

## Data Availability

The data that support the findings of this study are available on request from the corresponding author. The data are not publicly available due to privacy or ethical restrictions.

## Abbreviations

TIM-3: T cell immunoglobulin and mucin-domain containing protein 3
PD-L1: Programmed death-ligand 1
ICC: Intrahepatic cholangiocarcinoma
NSCLC: Non-small cell lung cancer
GC: Gastric cancer
HCC: Hepatocellular carcinoma
GC: Gemcitabine plus cisplatin
ICIs: Immune checkpoint inhibitors
CAFs: Cancer-associated fibroblasts
TAMs: Tumor-associated macrophages
TILs: Tumor-infiltrating lymphocytes
TME: Tumor microenvironment
mOS: Median overall survival
mDFS: Median disease-free survival
CPS: Combined positive score
IHC: Immunohistochemistry
SD: Standard deviation
HR: Hazard ratios
CI: 95% confidence intervals
NLR: neutrophil-to-lymphocyte ratio
EMT: Epithelial‑mesenchymal transition
